# The Gold-headed Cane

**Published:** 1860-09

**Authors:** 


The Gold-headed Cane. III.—In continuing our notices of the autobiography of the gold headed cane, we come to its third possessor, in the person of Dr. Askew, who had always entertained for Dr. Mead a sort of filial veneration. Of the early scholarship of Dr. Askew some idea may be formed, from the fact of his having dedicated, when a student at Leyden, his specimen of an edition of Æschylus to Dr. Mead. He was, also, a large collector of books; and at the sale of the library of his distinguished predecessor and friend, just named, he was one of the most eager of the *emptores literarii.* Not content with possessing himself as much as possible of his Greek manuscripts, books, statues, and other curiosities, he contrived to preserve the lineaments and perpetuate the memory of Dr. Mead, by procuring the eminent sculptor Roubiliac to make a bust of him. The gold-headed cane avers it to be a life-like work of art. Dr. Askew was so pleased with the execution of the bust that, although he had previously agreed with the sculptor for £50 sterling, or $200, he offered him double this sum, but, to his astonishment, "the sordid Frenchman exclaimed it was not enough, and actually sent in a bill for £108 2 shillings." This demand, even to the odd shilling, was paid; and Dr. Askew sent the receipt to Hogarth to produce at the next meeting of artists. Little did Roubiliac think of the danger he was in of being "damned to everlasting fame," by falling into the hands, or rather forming a subject for the pencil, of the satirical painter. But we anticipate.

Dr. Askew was absent three years from England, during which period he traveled in different countries, at a time when traveling was an unusual thing, and not, as at the present day, when English and American women visit Egypt and the Nile, traverse the desert and roam over Palestine, as, but a few years before, their mothers had thought it quite a feat to do over France and Italy. He visited Hungary, and resided at Athens and Constantinople. One of the immediate results of his travels was the excellent opportunity it afforded him of collecting books, manuscripts, and inscriptions. At Paris, on his way home from his Eastern expedition, he laid the foundation of his library, which became afterwards so celebrated. Of whom among the ancient and wealthy physicians, even of London and Paris, not to speak of Boston, New York and Philadelphia, can the biographer draw at the present day such a picture as the following, in matters of refined hospitality, book collections, and scholarship?

"Our house in Queen Square," says the gold-headed cane, "was crammed full of books. We could dispense with no more. Our passages were full, even our very garrets overflowed; and the wags of the day used to say, that the half of the square itself would have done so, before the book appetite of Dr. Askew would have been satiated.

"We saw a great deal of company, attracted as well by the abundant luxuries with which my master's table was furnished, as by the classical conversations and learned accounts of curiosities which he had brought with him from his very interesting travels in Greece. Among the literary people who were most frequently there, I may mention Archbishop Markham, Sir William Jones, Dr. Tanner, Demosthenes Taylor, and Dr. Parr. By these distinguished persons Dr. Askew was considered as a scholar of refined taste, sound knowledge, and indefatigable research into everything connected with Grecian and Roman learning. As a collector of books he was the first who brought bibliomania into fashion; and no one exhibited his various treasures better than himself. The eager delight with which he produced his rare editions, his large paper copies, his *glistering gems* and *covetable tomes*, would have raised him high in the estimation of the Roxburghe Club."

Most of the foreigners who came to London were received at Dr. Askew's house. Among them was a Chinese, named Chequa, who, as a mark of gratitude for the attention and kindness he had received, requested permission to take, before his departure from England, a model of the doctor in his robes. This was readily granted, and we, says the gold-headed cane, sat to the stranger.*

*Dr. Heberden.*—Among the contemporaries of Dr. Askew was a physician who had settled in London two years before him, and who rapidly got into great business, which he followed with unremitting attention above thirty years. This person was Dr. William Heberden, who was the first to suggest to the College of Physicians the publication of its Medical Transactions, to which he contributed several papers, as he did to the Philosophical Transactions of the Royal Society. His most noticeable and truly memorable work was, "Commentaries on the History and Cure of Diseases," published in the seventy-third year of his age.

"But though rendered eminent by his talents as a physician, he conferred a more valuable and permanent lustre on his profession by the worth and excellence of his private character. From his early youth Dr. William Heberden had entertained a deep sense of religion, a consummate love of virtue, an ardent thirst after knowledge, and an earnest desire to promote the welfare and happiness of all mankind. By these qualities, accompanied with great sweetness of manners, he acquired the love and esteem of all good men, in a degree which perhaps very few have experienced; and after passing an active life with the uniform testimony of a good conscience, he became a distinguished example of its influence, in the cheerfulness and serenity of his latest age."

An incident is related in the little volume now before us, happily illustrative of Dr. Heberden's practical piety. Many would have given advice not to publish an obnoxious treatise, but very few would have been willing to back this advice by a pecuniary grant out of their own private purses. After the death of Dr. Conyers Middleton, who was the author of an attack on the dignity of physic, so successfully repelled by Dr. Mead, his widow called on Dr. Heberden with a view of consulting him respecting a MS. treatise of her late husband on the inefficacy of prayer.

"Heberden having perused the MS., told the lady that, though the work might be deemed worthy of the learning of her departed husband, its tendency was by no means creditable to his principles, and would be injurious to his memory; but as the matter pressed he would ascertain what a publisher might be disposed to give for the copyright. This he accordingly did; and having found that £150 sterling ($600) might

* The model is about 12 inches high, and is of unbaked potter's clay.

be procured, he himself paid the widow £200 ($800,) and consigned the MS. to the flames."

The gold-headed cane, after the death of Dr. Askew in 1774, passed from his hands into those of Dr. David Pitcairn. There were three Pitcairns eminent in medicine. Archibald was made a professor of physic at Leyden, toward the close of the seventeenth century (1691.) He ranks among the physico-mathematical school, and is author of several dissertations, viz., on the circulation of the blood; the quantity of the blood; the motion of the stomach; the problem of inventions; circulation of the blood in both animals and embryons; concerning the cure of fevers by evacuation; acids and alkalies in the cure of distempers, etc. etc. Dr. Wm. Pitcairn took his degree of doctor of medicine at Oxford, on the day (April 13, 1749,) when the Radcliffe Library was opened with great solemnity. In the following year his name was transferred from the list of licentiates into that of the fellows of London College of Physicians. He practiced physic for nearly half a century, and was for several years president of the college.

Dr. David Pitcairn, successor to the possession of the gold-headed cane, was elected physician to St. Bartholomew's Hospital before he took his doctor's degree at Cambridge. His private medical practice dates from 1780. By the death of Dr. Warren he was placed at the head of his profession in London. He resided many years in Lincoln's Inn Fields, and was early admitted a Fellow of the Royal and Antiquarian Societies.

"I will endeavor," says our gold-headed autobiographer, "to describe one of the most remarkable evenings spent at a meeting of the first of these learned bodies.

"In the time of Dr. Mead, the Royal Society met in one of the Professor's rooms at Gresham College; and many of the members used to dine at Pontac's in Abchurch Lane. The house was kept by a Frenchman who had been cook to Mr. Pontac, president of the parliament of Bourdeaux; and who, from respect to the memory of his master, hung up his effigies as the outward sign of his place of entertainment. Since the apartments in Somerset House were allotted to the meetings of that scientific body, *the Club*, which consists of the more select of the Society, have for many years dined at the neighboring tavern—the Crown and Anchor—where, at half-past five o'clock, on each Thursday previous to the sitting of the Society, you are sure of meeting with very indifferent cheer, but excellent company. On the 7th of April, 1791, I accompanied Dr. Pitcairn to the tavern, and met there Prince Ponitowsky, who had been invited as a guest. Sir Joseph Banks was in the chair. His highness appeared about fifty, had a good face, was of middling stature, was dressed in black, had the order of Malta in his button-hole, and wore his hair in a round curl."

Our wooden narrator becomes garrulous, and we shall pass over some of its small-talk, including the details of a dinner given by the Prince, who was brother to the king of Poland. His highness repeated a story which he had heard from the Archbishop of Canterbury:—

"It related to a dramatic writer whose play had been a good deal applauded, and who was informed that on a particular night a great philosopher and mathematician was to be present at its performance. 'This,' said the author, 'is the man for me. I shall long to hear what he says of my play. The opinion of such a judge will be really worth having.' The mathematician took his seat in the centre of the pit; and when the performance was over, the author was anxious to have his opinion of the piece. 'I find,' said the philosopher, 'that such an actress has pronounced 3284 words, and that such an actor has pronounced

2864,' etc.; and this was the only reply that the mortified dramatist could obtain."

The following, though it may seem to be extravagant and out of nature, is not without its pendants in real life:—

"The Prince concluded his amusing anecdotes, and related to us that one of his brothers had engaged a Frenchman as a pastry-cook, but who was so drunken a fellow that a sentinel was always placed at the door to prevent his getting strong liquors before he had finished his work. At length, however, his frequent intoxication became intolerable, and it was necessary to discard him. He went to Dantzick, where he found a vessel bound to St. Petersburg, in which he embarked; and on his arrival in that city, accidentally heard of a nobleman, near Moscow, who was in want of a preceptor for his son. The *patissier* offered his services, was accepted, and traveled in an elegant coach to his destination. Of Italian, which he was to teach, he knew not a word; but being a native of Provence, he spoke the dialect of that part of France. This he taught to his pupil, and was for some time in great credit. But the nobleman having at length a visitor who spoke Italian, the impostor was detected, and he was ignominiously driven out of the family. For some months he rambled about Tartary, and lived on the hospitality of different hordes; but, after an absence of more than two years, finding his way back into Poland, he threw himself at the feet of his old master, and was again taken into service, upon promising better behavior in future."

The English reviewers sometimes twit American travelers for their over-readiness in publishing details of family life and private conversations in houses in which they have been received as visitors and guests. It would seem that this infirmity used to prevail among our older cousins themselves, nor have they entirely lost the habit at the present time. The Prince did not think very favorably of the English writing travelers, "*ces gouverneurs*," as he called them, who eagerly catch up everything they hear in conversation, for the sake of printing it. "The English Minister at Warsaw had observed to him, that he found himself oftentimes situated awkwardly enough with his raw young countrymen; but that this was nothing when compared with the trouble he had when they came accompanied with a traveling pedant as a tutor."

The conversation having turned on Russia, the Prince spoke of a certain courtier, who, when Biron was disgraced, said: "Ay, that fellow was the cause of my losing two of my teeth." "How so?" said somebody. "Why, because a dentist came here whom he patronized; and, in order to pay my court to Biron, I sent for that man to draw two of my teeth!"

To return from these *facetiæ* to Dr. Pitcairn. This eminent physician "was perfectly candid in his opinions; and very frank in acknowledging the extent of his confidence in the efficacy of medicine. To a young friend, who had very recently graduated, and who had accompanied him from London to visit a lady, ill of consumption, in the country, and who, on their return, was expressing his surprise at the apparent inertness of the prescription which had been left behind,—it being nothing more than an infusion of roses with a little additional mineral acid,—he made this reply: 'The last thing a physician learns, in the course of his experience, is to know when to do nothing, but quietly to wait, and allow nature and time to have fair play in checking the progress of disease, and gradually restoring the strength and health of the patient.'" His manner was simple, gentle, and dignified; and his kindness of heart led him to give more than mere routine attendance on his patients, by whom he was, in consequence, much beloved. "He associated much with gentlemen of the law, and had a taste for the fine arts." "His person was tall and erect; his countenance during youth was a model of manly beauty, and even in advanced life he was remarkably handsome."

It was reserved for Pitcairn, who had never published anything himself, to enlighten the profession by furnishing, in his own person, an example of a disease which, at the time, was regarded as entirely new, but which we would now speak of as one of secondary croup or diphtheria. Besides the ordinary symptoms of croup, Pitcairn had suffered from painful deglutition and partial swelling of the fauces. In its progress the voice was changed, became altogether suppressed, and the disease terminated in suffocation.

Dr. Baillie succeeded Dr. Pitcairn in the possession of the gold-headed cane, and to no small extent also in his practice. These two eminent physicians had long been on terms of the warmest friendship. But the time was passing away in which externals carried with them any weight as symbolic of professional position or merit. The autobiographer laments this change in the following terms:—

"When I passed from the hands of Pitcairn into the possession of Dr. Baillie, I ceased to be any longer a necessary appendage of the profession, and consequently the opportunities I enjoyed of seeing the world, or even of knowing much about the state of physic, were very greatly abridged, and but of rare occurrence. Once only was I introduced into a large party. It was on a Sunday evening, when I was taken to one of the scientific meetings held at the house of Sir Joseph Banks, in Soho Square. How different from the gay conversaziones in Ormond Street, in the spacious library of Dr. Mead, filled with splendid books, and ornamented with antiques of the most costly description! On entering the house of Sir Joseph, I was ushered up a sort of back staircase, and introduced into two gloomy apartments, in the farther corner of the first of which sat the President of the Royal Society, wearing the red ribbon of the Order of the Bath, in a gouty chair. Here I was passed from one to the other, and considered rather as a curious relic than regarded, as I was wont to be, as the support and ornament of the faculty. My only consolation arose, as I was handed about, from the observation, which it was impossible not to make, that among the philosophers present there was a great proportion of medical men, who examined me, as may be supposed, with more than ordinary interest.

"From what had been stated of the condition to which I was now reduced, it will be inferred that it was chiefly from the position which I occupied in the corner of the room in which Dr. Baillie received his patients at home, that I became at all acquainted with what was going on in medicine.

"My present was the very reverse of my early master, Dr. Radcliffe. In person, Dr. Baillie was considerably below the middle size, with a countenance rather plain than prepossessing, a Scotch dialect, and blunt manners. Than his first address nothing could be less imposing; and yet before he had been in company with you for five minutes, he would have convinced you that he was one of the most sensible, clear-headed physicians you had ever listened to."

A few lines on his early training in medical studies will properly precede our remarks on the professional character of Dr. Baillie. His mother was the sister of John Hunter. From the University of Glasgow he went, in 1780, to Baliol College, Oxford, where he graduated: he then settled in London under the immediate superintendence of his other maternal uncle, Dr. William Hunter.

"Following the example of his distinguished relations, he became himself a teacher of anatomy, in 1785; and he continued to lecture for nearly twenty years. In delivering his lectures, he expressed himself with great clearness, and conveyed his information to his pupils in the most simple and intelligible language. For this talent he was greatly indebted to the assiduous instruction of his uncle, who spared no pains in cultivating in his young pupil a habit of ready and exact explanation; and was accustomed to teach him in this manner: 'Matthew, do you know anything of to-day's lecture.' demanded Dr. Hunter of his nephew. 'Yes, sir, I hope I do.' 'Well, then, demonstrate to me!' 'I will go and fetch the preparation, sir!' 'Oh, no, Matthew, if you know the subject really, you will know it whether the preparation be absent or present.' Dr. Hunter then stood with his back to the fire, and his nephew demonstrated. Thus was the young student encouraged by approbation and assistance, or immediately convicted of loose and inaccurate information."

Doctor Baillie, from his habit of public lecturing, had acquired two great advantages: first, a minute and accurate knowledge of the structure of the human body, which is not always possessed even by eminent physicians; and, secondly, a distinct arrangement in what has been appropriately called the art of *statement.* His examination of a patient, for the purpose of learning the symptoms of the complaint, was short, and seemingly in haste, judging from the small number of questions put; but in conversing on the case with the physician whom he met in consultation, his views expressed in brief, although short, were clear. It was only, however, when the friends and relations of the patient were admitted, and he proceeded to communicate to them the result of the consultation that he appeared to the fullest advantage. He then gave a short practical lecture, not merely on the symptoms of the disease, but on its nature generally, in which all that was known on the subject was brought to bear on the individual case; and in doing this, his utterance was so deliberate that it was easy to follow him. It is very obvious that if we admit this practice to be worthy of general imitation, it must be carried out with considerable judgment, and in a manner and to an extent proportioned to the intelligence and good sense of the persons addressed on the occasion. It will, of course, be objected to by the racing or very busy practitioner, who hurries from house to house, not giving himself time to make the requisite examination of the patient, nor, still less, to give the desired explanations.

Dr. Baillie was not a routinist. His maxim was, that the most successful treatment of patients depended upon the exertion of sagacity or good common sense, guided by a competent professional knowledge, and not by following strictly the rules of practice laid down in books, even by men of the greatest talents and experience. There must be an endless variety in diseases, growing out of a corresponding variety of constitutions; and a necessity for continual modifications of practice, to be made by a judicious physician. In his view of the case of a patient, Baillie selected the leading features, and, neglecting all minor details, he abstained from touching on anything which was merely ingenious, not to say subtle, or far-fetched. "Hence, in the treatment of disease, he was not fertile in expedients, but aimed at the fulfillment of a few leading indications, by the employment of the simplest means; if these failed, he was often at a loss what to do next, and had not the talent, for which some are distinguished, of varying his prescription every day, so as to retain the confidence and keep alive the expectation of the patient." After writing a prescription, he read it over with great care and consideration, for fear of having committed a mistake.

In the hurry of great business, his day's work, as he used to say, amounted to seventeen hours. Baillie was sometimes irritable, and betrayed a want of patience in hearing the tiresome details of an unimportant story. His reply to a lady, who had wound up a wearisome detail of her ailments, just before her going to the opera, by asking him whether, on her return, she might eat some oysters, "Yes, Ma'am, shells and all," has often been told, with sometimes the substitution of the name of Abernethy for that of Baillie. During his latter years when he had retired from all but consultation, he was deliberate, tolerant, and willing to listen to whatever was said to him by the patient. Contrary to the predictions of his early contemporaries, and, we may add, a still pervading belief among professional men, Baillie acquired business early by the credit of his work on Morbid Anatomy, from the date of its publication, in 1793. The most eminent men in surgery and midwifery, both in London and Paris, have been lecturers on Anatomy, and we see from the example of Baillie that a similar course of teaching is decidedly advantageous in the practice of medicine. But let it be well understood, that a knowledge of anatomy is only an aid, and never a substitute for a careful study of therapeutics and clinical observation.

In imitation of the immortal Harvey, Dr. Baillie gave, in his lifetime, the whole of his most valuable collection of anatomical preparations to the College of Physicians, and six hundred pounds for the preservation of the same.

Dr. Baillie had "looked through Nature up to Nature's God," and "the promises of the gospel, on the conditions explained by our Redeemer, were his humble but confident hope in life, and his consolation in death."

